# Molecular detection of human enteric viruses circulating among children with acute gastroenteritis in Valencia, Venezuela, before rotavirus vaccine implementation

**DOI:** 10.1186/s13099-018-0232-2

**Published:** 2018-02-22

**Authors:** Ana C. Alcalá, Kriss Pérez, Ruth Blanco, Rosabel González, Juan E. Ludert, Ferdinando Liprandi, Esmeralda Vizzi

**Affiliations:** 1 Laboratorio de Biología de Virus, Centro de Microbiología y Biología Celular, Instituto Venezolano de Investigaciones Científicas (IVIC), Apdo. 21827, Caracas, 1020 Venezuela; 2 Instituto Autónomo de Biomedicina Dr. Jacinto Convit-MPPS, Caracas, Venezuela; 3 Departamento de Infectómica y Patogénesis Molecular, Centro de Investigación y Estudios Avanzados del Instituto Politécnico Nacional, Mexico, D.F. Mexico; 4 Present Address: Departamento de Medicina Molecular y Bioprocesos, Instituto de Biotecnología, Universidad Nacional Autónoma de México (UNAM), Mexico, D.F. Mexico

**Keywords:** Acute gastroenteritis, Children, Enteric viruses, Prevalence, Venezuela

## Abstract

**Background:**

The role of rotavirus as main etiologic agent of diarrhea has been well documented worldwide, including in Venezuela. However, information about the prevalence of gastrointestinal viruses such as calicivirus, adenovirus and astrovirus is limited and the contribution of other agents as Aichi virus and klassevirus is largely unknown. To explore the etiological spectrum of diarrhea associated with agents other than rotaviruses, 227 stool samples from children under 5 years old with acute gastroenteritis, collected in Valencia (Venezuela) from 2001 to 2005, and previously tested as rotavirus-negative, were analyzed for caliciviruses, adenoviruses, astroviruses, Aichi viruses, klasseviruses, picobirnaviruses and enteroviruses by specific RT-PCRs.

**Results:**

At least one viral agent was detected in 134 (59%) of the samples analyzed, mainly from children under 24 months of age and most of them belonging to the lowest socioeconomic status. Overall, enterovirus was identified as the most common viral agent (37.9%), followed by calicivirus (23.3%), adenovirus (11.5%), astrovirus (3.5%), klassevirus (1.3%) and Aichi virus (0.4%), while no picobirnavirus was detected. Klasseviruses were found during 2004 and 2005 and Aichi viruses only in 2005, indicating their circulation in Venezuela; meanwhile, the rest of the viruses were detected during the whole study period. Coinfections with two or more viruses were found in 39 (29.1%) of the infected children, most under 24 months of age. Adenovirus was involved as the coinfecting agent in at least 46.9% of the cases, but no differences concerning socio-demographic variables were observed between the coinfected and the single infected children.

**Conclusions:**

The results show that various enteric viruses, including enteroviruses, caliciviruses and adenoviruses, accounted for a significant proportion of infantile diarrhea cases in Venezuela before rotavirus vaccine implementation. In addition, emerging viruses as Aichi virus and klassevirus were found, indicating the need to continue monitoring their spreading into the communities. Efforts are needed to develop more accurate methods to identify the major causes of diarrhea and to provide tools for more effective preventive measures.

## Background

Acute gastroenteritis (AGE) in children is one of the most significant diseases, causing morbidity and mortality worldwide [1, 2]. Although the improvement in sanitation and prevention strategies has determined a substantial reduction in the mortality rate for diarrhea from 15% in 2008, to about 9% in 2015, equivalent to 500,000 deaths among children less than 5 years old, infectious diarrheas are still an important public health concern, both in resource-poor settings and industrialized countries [2, 3].

Viruses are recognized as a major cause of severe AGE, particularly in children. Rotaviruses (RV) are the main cause of mortality due to diarrhea in those under 5 years old, preventable with the vaccination [4, 5]. Yet, despite a significant reduction after RV vaccine introduction in 2006, hospitalizations for infantile AGE of viral etiology continued to be reported [6–9]. The increasing use of more powerful diagnostic systems in the last few years, as the conventional polymerase chain reaction (PCR) or high-throughput technologies, for the amplification and identification of virus genomes in stool samples, has resized the study of the agents involved in childhood AGE [7, 10, 11], and changed significantly the pathogen spectrum of community-acquired gastroenteritis [6, 7, 12, 13].

Other viruses considered of clinical importance and frequently associated with diarrhea mortality are the human caliciviruses (HuCVs), members of the family *Caliciviridae*, which have acquired importance, especially after RV vaccine implementation [6, 10, 14]. Human adenoviruses (HAdVs), members of the family *Adenoviridae*, are often reported as the second or third cause of infantile diarrhea, both sporadic or outbreak associated, and cause a wide range of disease symptoms [14, 15]. Finally, human astroviruses (HAstVs), of the family *Astroviridae*, which affect predominantly children under 2 years of age, have been involved in 0.5–15% of diarrheal outbreaks associated to severe pediatric cases [16–18].

On the other hand, the clinical importance of viruses belonging to the wide family *Picornaviridae*, such as enterovirus (EV), Aichi virus (AiV) and klassevirus/salivirus (KV), is up-to-date unclear, with those viruses presumably playing a rather minor epidemiological role in diarrhea [14, 19–22]. Some subgroups of EVs have been involved as causative of at least 3.4% of AGE of unknown etiology [23]. Similarly, AiV, of the genus *Kobuvirus*, was initially described as cause of oyster-associated non-bacterial gastroenteritis in human [22], and later associated with AGE, reaching detection rates between 0.5 and 0.9% in Europe, and up to 4% in Asia and Africa [20, 24]. AiVs were recovered during a study from a major river polluted with sewage discharges in Caracas (Venezuela) during 2007–2008 [25], but its impact on the burden of AGE in Venezuela is unknown. Finally, KVs, discovered in human stool and sewage [22], have been significantly associated with pediatric diarrhea in different countries, especially in children less than 3 years old, with a frequency ranging from 0.1 to 8.7% [26–28].

Because the information about viruses different from RV associated with diarrhea in Venezuela is limited, the present study was aimed to determine the incidence of infections caused by other conventional gastroenteritis viruses before RV vaccine implementation, and to investigate the contribution of AiV and KV to diarrheal diseases, until now unknown in Venezuela. For this purpose, children less than 5 years old with diarrhea attended at a large public hospital in Valencia City, over a 5-year period (2001–2005), were studied using molecular detection assays.

## Methods

### Study design

The study included stool samples collected from children with AGE under 5 years old, attended during the years 2001–2005 in the city of Valencia, Carabobo State (Venezuela), as part of a RV diarrhea surveillance program conducted at the *Hospital de Niños ‘‘Dr. Jorge Lizarraga’’* of the *Ciudad Hospitalaria ‘‘Dr. Enrique Tejera’*’ (CHET) described previously [29].

AGE was defined as three or more liquid stools over a 24-h period and for not over 14 days. To determine the epidemiologic and clinical characteristics of the AGE, information from the clinical history and the physical examination were collected: age, gender, nutritional status and type of treatment (outpatient or inpatient hospital based) were recorded for each case and used as measurement instrument for the severity of the community-acquired AGE, together with the estimation of dehydration, assessed according to WHO criteria [30]. Inpatient treatment was defined as the admission to either the emergency room for a short stay to receive oral rehydration therapy (< 24 h) or to the regular pediatric wards of the hospital for longer time [31]. The socioeconomic status was determined by a modified Graffar methodology [32].

### Sample collection

From a total of 13,026 fecal diarrhea specimens obtained from the enrolled children within 48 h following admission, 227 were randomly selected from RV negative-tested samples. All the samples had been systematically examined for the presence of RV antigen, bacteria and parasites as previously described [29, 33] and resulted negative for all of them. All samples were stored at − 80 °C until processed.

### Nucleic acid extraction

Fecal suspensions (10% w/v in phosphate buffer saline) were prepared from each stool sample, vortexed, and clarified by centrifugation at 10,000*g* for 10 min. Viral RNA/DNA was extracted simultaneously from 200 µl of supernatant, using the QIAamp MinElute Virus Spin Kit (QIAGEN, Hilden, Germany), based in a spin-column procedure, and following the manufacturer’s instructions. Briefly, samples were lysed in the presence of QIAGEN Protease and Buffer AL containing RNA carrier provided by the kit. Ethanol absolute (Merck, KGaA, Darmstadt, Germany) was added to the sample that was then transferred onto a QIAamp MinElute column, where the viral nucleic acids were adsorbed onto the silica-gel membrane. Wash buffers were used to remove impurities by centrifugation, and finally, the viral nucleic acids were eluted in 50 µl of Buffer AVE (provided), for use in amplification reactions or storage at − 70 °C.

### Reverse transcription (RT)

Screening for the presence of RNA viruses, such as HuCV, HAstV, AiV, KV, EV and human picobirnavirus (HPBV), was conducted firstly by RT reaction, as follows: the extracted RNA was denatured and then reverse transcribed with random primers (0.02 µg) using M-MLV reverse transcriptase (200 U) and deoxynucleoside triphosphate mix (0.2 mM), RNasin (40 U) (Invitrogen, Carlsbad, California, USA) in reverse transcription buffer to a final volume of 50 µl. The mixture was incubated at 37 °C for 1 h followed by incubation at 70 °C for 15 min, to obtain cDNA.

### Polymerase chain reaction (PCR)

Single PCR reactions were performed from 5 µl of extracted DNA (for HAdV detection), or cDNA (for RNA viruses), using a selected combination of oligonucleotide primers specific for each virus previously described [22, 34–40] at a final concentration of 0.2 µM each one. Two additional degenerated primers were designed for this study by multiple alignments, leading to broad target specificity for HuCV (290YM) and HAstV (MON394d) (Table 1). Cycling conditions used were adapted as shown in Table 1. All PCR reactions were done in a final volume of 50 µl and the PCR products were analyzed by agarose gel electrophoresis and ethidium bromide staining.

**Table 1:** Oligonucleotide primers and amplification conditions used in this study for the molecular detection of gastroenteritis viruses

Virus	Target region	Rounds of PCR	Sense	Primer name	Sequence 5′–3′	Cycling protocol^1^	Nucleotide position	Amplicon size (bp)	Reference
Calicivirus	RNA-dependent RNA polymerase	1st	−	289H	TGACGATTTCATCATCACCATA		4865–4886^a^		34
			−	289I	TGACGATTTCATCATCCCCGTA	A	4865–4886^a^	319	
			+	290YM	GATTACTCCAGGTGGGAYTCMAC		4568–4590^a^		In this study
Adenovirus	Hexon	1st	+	hexAA1885	GCCGCAGTGGTCTTACATGCACATC	B	18,858–18,883^b^	301	35
			−	hexAA1913	CAGCACGCCGCGGATGTCAAAGT		19,136–19,158		
Astrovirus	ORF-1	1st	+	MON340	CGTCATTATTTGTTGTCATACT	C	1182–1203	289	36
			−	MON348	ACATGTGCTGCTGTTACTATG		1450–1470		
		2nd	+	MON394d	GARATCCGTGATGCTAATGG	D	1250–1269	220	In this study
			−	MON348	ACATGTGCTGCTGTTACTATG		1450–1470		37
Aichi virus	3C-3D	1st	+	6261	ACACTCCCACCTCCCGCCAGTA	E	6261–6282^c^	519	38
			−	6779	GGAAGAGCTGGGTGTCAAGA		6760–6779		
Klassevirus	2C	1st	+	LG0098	CGTCAGGGTGTTCGTGATTA	F	4463–4482	345	27
			−	LG0093	AGAGAGAGCTGTGGAGTAATTAGTA		4783–4807		
Enterovirus	5′NTR^2^	1st	+	EV1	CGGCCCCTGAATGCGGC	G	454–470	194	39
			−	EV2	CACCGGATGGCCAATCCA		630–647		
Picobirnavirus			+	PicoB25	TGGTGTGGATGTTTC		665–679^d^	201	
	RNA-dependent RNA polymerase	Multiplex PCR	−	PicoB43	ARTGYT GGTCGAACTT	H	850–865^d^		40
			+	PicoB23	CGGTATGGATGTTTC		685–699^e^	369	
			−	PicoB24	AAGCGAGCCCATGTA		1039–1053^e^		

^1^Cycling conditions for the PCRs were as follows: A = 94 °C for 2 min, 40 cycles of 94 °C for 30 s, 50 °C for 30 s, 72 °C for 1 min, and a final elongation at 72 °C for 10 min; B = 94 °C for 4 min, 40 cycles of 92 °C for 1.5 min, 55 °C for 1.5 min, 72 °C for 2 min, and final elongation at 72 °C for 10 min; C = 94 °C for 5 min, 40 cycles of 94 °C for 30 s, 50 °C for 30 s, 72 °C for 30 s; and final elongation at 72 °C for 10 min; D = 94 °C for 2 min, 30 cycles of 94 °C for 30 s, 50 °C for 30 s, 72 °C for 30 s, and final elongation at 72 °C for 10 min; E = 95 °C for 1 min, 40 cycles of 95 °C for 30 s, 55 °C for 30 s, 72 °C for 1 min and a final elongation at 72 °C for 10 min; F = 94 °C for 2 min, 40 cycles of 94 °C for 30 s, 56^◦^C for 30 s, 72 °C for 1 min, and final elongation at 72 °C for 10 min; G = 94 °C for 2 min, 40 cycles of 94 °C for 30 s, 60 °C for 30 s, 72 °C for 1 min, and a final elongation at 72 °C for 7 min. H = 94 °C for 3 min, 40 cycles of 94 °C for 1 min, 42 °C for 1 min, 72 °C for 1 min, and a final elongation at 72 °C for 10 min

^2^5′nontranslated region

^a^Numbering given according to positions in Norovirus GI, complete genome (NC_001959.2)

^b^Sequence position refers to the Ad2 hexon region. The primers used allow detecting the 47 human adenovirus serotypes

^c^Sequence position refers to Aichi virus genomic RNA, Ac. N. AB010145.1

^d^Sequence position refers to the 1-CHN-97 strain (Genogroup I)

^e^Sequence position refers to the 4-GA-91 strain (Genogroup II)

### Statistical analysis

Data were analysed for the comparisons of variables using 2 × 2 tables with χ^2^ test, or Fisher’s exact test (two-tailed, 95% confidence intervals) (Epi Info™ 7.1.4.0, CDC Atlanta, GA, USA). Student’s test was applied for comparisons of variable values. Tests were considered significant when p < 0.05.

## Results

### Prevalence of the viral infections

**Fig. 1:**
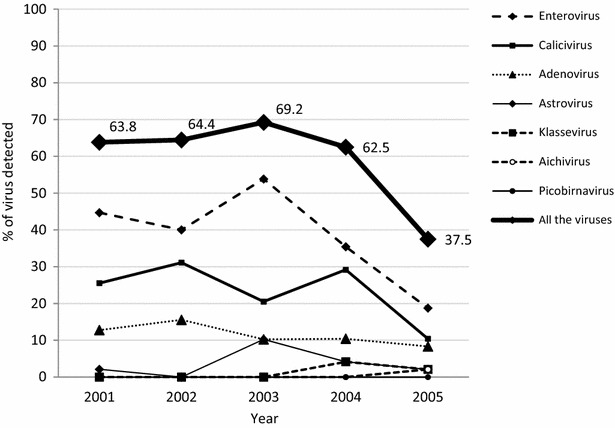
Temporal variation of the positivity rate for gastroenteritis viruses in children with diarrhea from Valencia, 2001–2005. The prevalence of viral infection among the 227 RV-negative children studied is indicated with the line marked with diamonds. A significant drop (p < 0.015) in the overall percentage of children infected was observed in 2005. The others drawn lines show the temporal detection rates for each single viral infection

Overall, the analysis by RT-PCR for HuCV, HAdV, HAstV, AiV, KV, EV and HPBV of the 227 selected stool samples negative for RV, enteropathogenic bacteria and parasitic infections, revealed the presence of at least one viral agent in 134 (59%) of them. The annual prevalence of viral infection fluctuated around an average of 65%, with comparable values between 2001 and 2004 (range 62.5–69.2%) and a significant drop (37.5%) in 2005 (p < 0.015) (Fig. 1).

### Detection rate and temporal variation of gastrointestinal viral agents

**Fig. 2:**
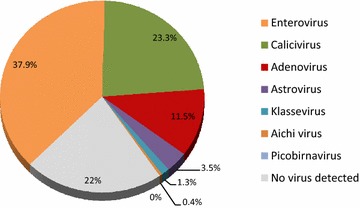
Distribution (%) of gastrointestinal viruses among 227 children with non-RV associated diarrhea from Valencia, 2001–2005

Figure 2 shows the detection rate for each single virus found in the stools of the 227 children studied from Valencia. EV was the most common etiological agent detected, present in 86 (37.9%) of the 227 samples, followed by HuCV in 53 (23.3%), HAdV in 26 (11.5%), HAstV in 8 (3.5%), KV in two (1.3%) and AiV in one (0.4%) (Fig. 2). No HPBV was detected.

As shown in Fig. 1, EV, HuCV, and HAdV were detected throughout the study period, while the finding of HAstV, KV and AiV was intermittent (particularly KV and AiV that emerged at the last years). A significant decrease (from 53.8 to 18.8%, p = 0.0006) in the detection rate for EV was observed between 2003 and 2005; a reduction was also observed for HuCV (from 29.2 to 10.4%, p = 0.021) from 2004 to 2005. No trend was observed for the other viruses (Fig. 1).

### Socio-demographic variables of all the children studied

#### Age

**Fig. 3:**
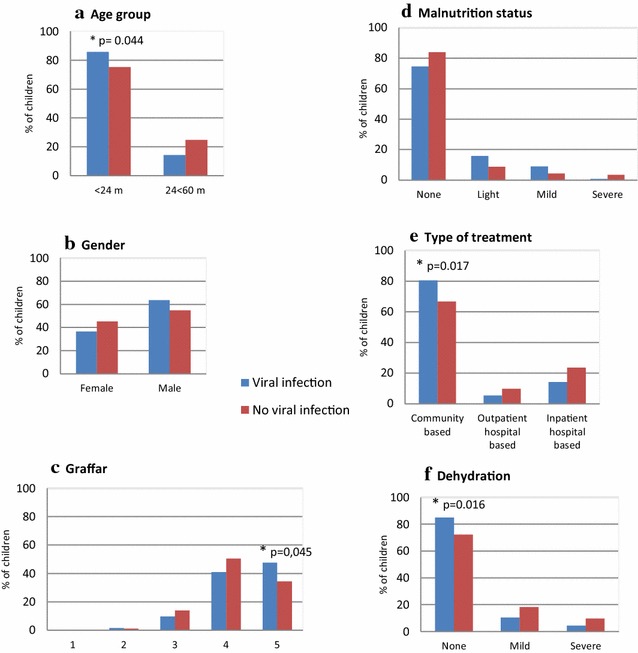
Distribution (%) of virus positive and negative children in according to demographic and clinical characteristics. A comparison between the proportion of children with viral infection (n = 134, histogram bars in blue color) and those without any virus detectable with the specific primers used in this study (n = 93, in red color) is shown. The variables shown are: **a** age group, **b** gender, **c** Graffar, **d** malnutrition status, **e** type of treatment, and **f** dehydration. Significant differences among groups are indicated with asterisk (*), including “p” value

The median age of the 227 children studied was of 11 months (range 1–58). No significant difference was observed in the median age of the 134 virus infected children when compared with the 93 children who resulted negative for all viral agents studied (diarrhea of unknown etiology) (13 vs. 10 months, p = 0.218). For the children under 24 months of age, the proportion of positive samples for viruses was significantly higher than that of negative samples (85.8% vs. 75.3%) (p = 0.044) (Fig. 3a).

#### Gender

A predominance of male over female was observed among the children positive, as well as among those negative children for viral infection (Fig. 3b), and the differences within genders were not significant (p > 0.05).

#### Socioeconomic level (Graffar)

The proportion of children from the lowest socioeconomic level (Graffar 5) was significantly higher among the positive for viral detection than among those negative (47.8% vs. 34.4%, p = 0.045) (Fig. 3c).

#### Malnutrition status

Well-nourished children prevailed over those with some condition of malnourishment; nevertheless, no statistical significant differences (p > 0.05) were observed for infected or non-infected children in any status (Fig. 3d).

#### Type of treatment

The diarrhea caused by viruses was significantly more associated with outpatient episodes that did not require hospitalization than diarrhea episodes of unknown etiology, not caused by any of the viruses studied (80.6% vs. 66.7%, p = 0.017) (Fig. 3e).

#### Dehydration

The proportion of children who suffered a mild or severe form of dehydration was significantly lower among virus infected children than among children with no detectable virus (14.9% vs 28%, p = 0.016) (Fig. 3f).

### Incidence of single or mixed infection

Of the 134 children with diarrhea caused for any of the viruses studied, 95 (70.9%) resulted positive at a single enteric viral pathogen, and 39 (29%) suffered a mixed infection or coinfection (simultaneous detection of two or more viruses) (Table 2). The analysis of the socio-demographic variables and clinical parameters in relation with the severity of the AGE between single and mixed infected children did not reveal any significant difference (p > 0.05). Thus, age, gender, Graffar, nutritional status, dehydration and type of treatment of the children infected with a single virus were similar to those observed in children with coinfections (Table 2).

**Table 2:** Comparison of the demographic and clinical characteristics of children suffering single or mixed viral infections

	Single infection	Mixed infection
N. of children infected	95 (70.9)	39 (29.1)
Median age, months	13	11
Age group, months		
< 24	78 (82.1)	37 (94.9)
24–60	17 (17.9)	2 (5.1)
Gender		
Female	31 (32.6)	18 (46.2)
Male	64 (67.4)	21 (53.8)
Graffar socioeconomic level, n. (%)		
1	–	–
2	1 (1.1)	1 (2.6)
3	9 (9.5)	4 (10.3)
4	39 (41.1)	16 (41)
5	46 (48.4)	18 (46.2)
Malnutrition status		
None	73 (76.8)	28 (71.8)
Light	13 (13.7)	7 (17.9)
Mild	9 (9.5)	3 (7.7)
Severe	–	1 (2.6)
Dehydration		
None	82 (86.3)	31 (79.5)
Mild	10 (10.5)	5 (12.8)
Severe	3 (3.2)	3 (7.7)
Type of treatment		
Outpatient	78 (82.1)	30 (76.9)
Inpatient	17 (17.9)	9 (23.1)

Data are n (%) of children studied. No significant difference (p > 0.05) related with these variables was observed. Data were analysed using χ^2^ or Fisher’s exact test (two-tailed, 95% confidence intervals) when the size sample was less than 5 (Epi Info™ 7.1.4.0, CDC Atlanta, GA, USA). The significance of the difference for the ages was calculated by Student’s test. The scale used for the Graffar socioeconomic level was based in a modified methodology described by Méndez Castellano et al. [32]

**Fig. 4:**
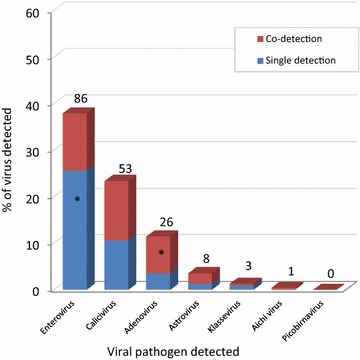
Proportion of gastrointestinal viruses involved in single or in coinfection in children with diarrhea from Valencia, 2001–2005. The figure shows the frequency of detection of each virus involved in single infection (only one virus detected, in blue color) or in co-infection (virus simultaneously detected together with one or more viruses, in red color). Numbers above the histogram bars indicate the total number of viruses detected (n = 177) among the 227 children studied. Significant differences (p < 0.05) compared to the rest of the groups are indicated with asterisk (*) inside the histograms

Figure 4 shows the proportion of gastrointestinal viruses involved in single or in coinfection during the AGE episodes. EVs were more frequently associated with single infection (58/86 strains detected, 67.4%, p = 0.003) than the other viruses. In contrast, HAdVs were significantly more involved in coinfections (18/26, 69.2%, p = 0.011) than in single infections. Finally, for HuCV and HAstV more than half of the infections were coinfections (54.7 and 62.5%, respectively) (Fig. 4).

The most frequent combination of coinfecting agents was given by HuCV/EV, which affected 15 of 39 children (38.5%) (Table 3). HAdV coinfected especially with EV (17.9%), HuCV (15.4%), both together (10.3%), or with HAstV (2.6%) (Table 3), which represent altogether 46.2% of the 39 mixed infections evaluated in this study. The only AiV strain found in this study was detected in mixed infection with a strain of HuCV (Table 2) in a severely dehydrated and malnourished child of 1 month old, living in poor conditions (Graffar 5), who required to be hospitalized.

**Table 3:** Viral agents involved in the 39 coinfections in children suffering diarrhea in Valencia, 2001–2005

Coinfection pattern	Number (%)
*Calicivirus* + *Enterovirus*	15 (38.5)
*Enterovirus* + *Adenovirus*	7 (17.9)
*Calicivirus* + *Adenovirus*	6 (15.4)
*Calicivirus* + *Adenovirus* + *Enterovirus*	4 (10.3)
*Calicivirus* + *Astrovirus*	2 (5.1)
*Calicivirus* + *Klassevirus*	1 (2.6)
*Calicivirus* + *Aichi virus*	1 (2.6)
*Adenovirus* + *Astrovirus*	1 (2.6)
*Astrovirus* + *Enterovirus*	2 (5.1)

Socio-demographic and clinical features of the children according to the gastrointestinal virus detected.

Table 4 shows the comparison of the socio-demographic and clinical parameters for the 95 children presenting only single virus infection. The analysis excluded HAstV and KV because of the low number of samples positive.

**Table 4:** Demographic and clinical characteristics of the children with single infection

	Enterovirus	Calicivirus	Adenovirus	Astrovirus	Klassevirus	Aichi virus	Picobirnavirus
N. of children infected	58	24	8	3	2	0	0
Median age, months	14	11	8	13	0.6	–	–
Age group, months							
< 24	45 (77.6)	21 (87.5)	7 (87.5)	3 (100)	2 (100)	–	–
24–60	13 (22.4)	3 (12.5)	1 (12.5)	–	–	–	–
Gender							
Female	16 (27.6)	9 (37.5)	3 (37.5)	2 (66.7)	1 (50)	–	–
Male	42 (72.4)	15 (62.5)	5 (62.5)	1 (33.3)	1 (50)	–	–
Graffar socioeconomic level							
1	–	–	–	–	–	–	–
2	–	1 (4.2)	–	–	–	–	–
3	6 (10.3)	2 (8.3)	1 (12.5)	–	–	–	–
4	21 (36.2)	11 (45.8)	4 (50)	1 (33.3)	2 (100)	–	–
5	31 (53.4)	10 (41.7)	3 (37.5)	2 (66.7)	–	–	–
Malnutrition status							
None	43 (74.1)	18 (75.0)	8 (100)	2 (66.7)	2 (100)	–	–
Light	7 (12.1)	5 (20.8)	–	1 (33.3)	–	–	–
Mild	8 (13.8)	1 (4.2)	–	–	–	–	–
Severe	–	–	–	–	–	–	–
Dehydration							
None	51 (87.9)	22 (91.7)	5 (62.5)	3 (100)	1 (50)	–	–
Mild	6 (10.3)	1 (4.2)	3 (37.5)	–	–	–	–
Severe	1 (1.7)	1 (4.2)	–	–	1 (50)	–	–
Type of treatment							
Outpatient	49 (84.5)	20 (83.3)	5 (62.5)	3 (100)	1 (100)	–	–
Inpatient	9 (15.5)	4 (16.7)	3 (37.5)	–	–	–	–

Data are n (%) of children studied. No significant difference (p > 0.05) related with these variables was observed. Data were analysed using χ^2^ or Fisher’s exact test (two-tailed, 95% confidence intervals) when the size sample was less than 5 (Epi Info™ 7.1.4.0, CDC Atlanta, GA, USA). The significance of the difference for the ages was calculated by Student’s test. The scale used for the Graffar socioeconomic level was based in a modified methodology described by Méndez Castellano et al. [32]

No significant difference was shown in the median age, gender, Graffar and nutritional status of the children affected, regardless of the infecting virus. Children less than 24 months prevailed over the oldest, and although the EV infected subjects were slightly in a higher percentage (22.4%) when compared with those infected with HuCV and HAdV, the differences were not significant (p > 0.05).

Regarding the variables related with the severity of the AGE, no virus was significantly associated with more severe dehydration or a greater number of inpatient episodes (p > 0.05).

## Discussion

The present study shows the epidemiology of viruses that caused pediatric AGE in Valencia (Venezuela) between 2001 and 2005 before the RV vaccine implementation. Although the population studied does not represent the entire epidemiological data of the viral diarrheal disease of this country, the results should provide a good estimation of the real impact of the viral AGE during the years 2001–2005 by causes other than RV.

The high prevalence of enteric virus found in this study is similar to that reported previously by others authors [12, 41, 42], and showed that EV, HuCV, HAdV, HAstV, AiV and KV accounted for a significant proportion of RV-negative AGE in this locality. The rate was lower than that shown by a Japanese study where multiplex assays including a larger number of target pathogens were applied [8], but it was higher than that described in European, Asian and African studies [17, 43–45], as well as that reported in a previous study performed during the year 2003 in Valencia City [46]. Of note, a fraction (41%) of the diarrhea cases here studied remained without a precise etiology, probably due to a low viral load, the presence of inhibitors in the samples or viruses not included in the assays. However, the relatively higher detection rate of viral agents reflects an increase of the diagnostic capabilities of the PCR-based assays used, although it could also depend on the population group studied, which included mostly children under 24 months of age, belonging to the lowest socioeconomic stratum (Graffar 5), living in the most precarious sanitary and dietary conditions, where the fecal–oral transmission of a wide range of pathogens was favored.

The significant higher frequency of viral infections, as well coinfections, shown here in children less than 24 months of age contrasts with previous data from Valencia where no age differences were observed in viral enteric infections [46]. It is instead in agreement with data obtained by others authors elsewhere [44, 47], and shows the highest susceptibility of the children to the viral infection during the early childhood, perhaps due to unsatisfactory protective immunity.

Previous data have reported that viral infections other than RV are clinically milder than the RV infection [44, 46, 48]. In this study, only RV-negative stool samples were included; therefore a comparison of the clinical conditions with children infected with RV could not be done. However, the data suggest that the infections by viruses such as EV, HuCV, HAdV, HAstV, AiV and KV would be mainly associated with less severe diarrheic episodes, not necessarily demanding medical intervention or hospitalizations.

This study demonstrates the contribution of EV and HuCV as important etiologic agents of viral AGE in the setting studied, both viruses found together in mixed infections in almost a quarter of the cases studied. The detection rate obtained for EV as single infecting agent was similar to that reported in a study carried out in Maracaibo (Venezuela) during 2008–2009 [49], and it was higher than that described in Thailand [23]. On the other hand, this rate was similar to the RV rate detection (24.5%) reported in another study carried out in Valencia City, during the same period [33]. Some serotypes of echovirus and coxsackievirus B have been described to be cause of diarrhea [50, 51]. It is noteworthy that the presence of Sabin vaccine-related strains in stool samples of diarrheic children could have caused an overestimation in the EV detection rate with the PCR assay used. In addition, EVs could be occasionally shed with the feces of patients suffering a broad spectrum of other non-enteric diseases, sometimes in prolonged way [20, 23, 50]. This would explain in part the relatively higher rate of EV found in this study in infected children older than 24 months than that of other viruses. Thus, case–control studies and further genotyping of the strains detected will be desirable, to better define the burden of EV as a cause of diarrheal disease.

The overall prevalence of HuCV observed in this study, the second most common causative agent of viral AGE, was comparable to that described by others among RV-negative samples from children with diarrhea in four distinct Thai regions under sentinel surveillance between 2006 and 2008 [52], and higher than that reported previously in Valencia City during the 2003 [46]. This prevalence indicates a greater ability of the primers used in the PCR assay to detect a broad diversity of strains. It ratifies also the need of monitoring the contribution of the HuCVs to the burden of the AGE after implementation of RV vaccination.

A significant observation in this study was also the relatively higher detection rate of HAdV infection, as compared to a previous study based on serologic assays from Valencia [46], and to reports from other continents [53–55] that suggest the existence of a geographic variability of the virus prevalence, as well as the important contribution of the HAdVs to the mixed infections. A similar rate of HAdV detection was reported from Korea during the years 2012–2013 [6], but it is noteworthy that the relative high prevalence for HAdV observed in this study could have also been determined by the presence in the stools of non-enteric types that could occasionally be excreted from respiratory source, and detected by the assay used, directed to amplify a conserved portion of the hexon-coding gene, common for all the HAdV. Thus, the molecular characterization is a crucial step to define the species of HAdV mainly involved in diarrhea and to understand the true contribution to the AGE. No information about the types of HAdV that have been circulating in Venezuela is available, but preliminary results indicate that most of the HAdV strains found in Valencia City during the same period were enteric types (Blanco R., personal communication).

HAstV were involved in a modest number of episodes, mainly in mixed infections with HuCV, HAdV and EV, which evolved as a mild form of AGE, similar to that reported by other studies [17, 41]. The HAstV detection rate found here was comparable with the data from a previous local study [46], and those from Lebanon, France and Germany [41, 44, 56].

Although AiV and KV have been associated with AGE in several continents [17, 24, 27, 28, 57–59], to our knowledge, there have not been reports of AiV and KV causing infections in Venezuelan human population. Unfortunately, the low rate of detection in this study did not allow to evaluate their relationship with socio-demographic and clinic variables, but their presence confirms the participation as agent of childhood diarrhea and the relatively recent introduction in Venezuela.

In this study were used primers directed to the most commonly described HPBVs of genogroup I and II [40], but no virus was found. Possibly, their high genomic diversity could have limited the detection with the RT-PCR assay available. Thus, additional efforts are required to optimize assays able to identify these and other uncommon viruses associated with AGE, as well the use of new technologies as virus microarray, sequence-independent amplification and sequencing of viral nucleic acids [7, 11, 22], to clarify their epidemiology and possible pathogenicity.

## Conclusions

This study demonstrated a high prevalence of enteropathogenic viruses other than RV in Venezuelan children suffering acute diarrhea, confirming the contribution of conventional enteric viruses in the pediatric AGE in this country. In addition, the presence of emergent viruses more recently described, such as AiV and KV is also described. Because the study included only diarrheic pediatric patients who received medical attention in Valencia City, the prevalence of virus infection reported here could represent an underestimation of the true rates of gastroenteritis associated viruses circulation in the population. Future studies should consider asymptomatic and self-limiting diarrhea cases. However, these results, obtained from five consecutive years, expand the knowledge about the spectrum of viral agents involved in acute community-acquired disease, and provide a baseline data for the molecular epidemiology study of these pathogens, which will be helpful for comparison with regional data obtained in post-RV vaccination era. Finally, they ratify the need for a long-term surveillance for such enteropathogenic viruses, following the implementation of RV vaccination, to better understand the participation of these agents in children AGE.

## Abbreviations

RT-PCR: reverse transcriptase polymerase chain reaction
AGE: acute gastroenteritis
RV: rotavirus
HuCV: human calicivirus
HAdV: human adenovirus
HAstV: human astrovirus
HPBV: human picobirnavirus
HBoV: human bocavirus
KV: klassevirus/salivirus
AiV: Aichi virus
EV: enteroviruses
CHET: *Ciudad Hospitalaria ‘‘Dr. Enrique Tejera”*
IVIC: Instituto Venezolano de Investigaciones Científicas
MMLV: Moloney Murine Leukemia Virus
cDNA: complementary DNA
WHO: World Health Organization
